# Efficacy of mavacamten in patients with hypertrophic cardiomyopathy and mid-ventricular obstruction: case series

**DOI:** 10.1093/ehjcr/ytaf229

**Published:** 2025-05-08

**Authors:** Valeria Rella, Denisa Muraru, Lia Crotti

**Affiliations:** Department of Cardiology, Cardiomyopathy Unit, Istituto Auxologico Italiano, IRCCS, Ospedale San Luca, Piazzale Brescia, 20, 20149 Milan, Italy; Department of Cardiology, Integrated Cardiovascular Imaging Department, Istituto Auxologico Italiano, IRCCS, Ospedale San Luca, Piazzale Brescia, 20, 20149 Milan, Italy; Department of Medicine and Surgery, University of Milano-Bicocca, Piazza dell'Ateneo Nuovo, 1, 20126 Milan, Italy; Department of Cardiology, Cardiomyopathy Unit, Istituto Auxologico Italiano, IRCCS, Ospedale San Luca, Piazzale Brescia, 20, 20149 Milan, Italy; Department of Medicine and Surgery, University of Milano-Bicocca, Piazza dell'Ateneo Nuovo, 1, 20126 Milan, Italy

**Keywords:** Hypertrophic cardiomyopathy, Mavacamten, Mid-ventricular obstruction, Case report

## Abstract

**Background:**

Mavacamten is a cardiac-specific myosin inhibitor approved for treatment of adults with hypertrophic cardiomyopathy (HCM) symptomatic for left ventricular outflow tract (LVOT) obstruction. Since obstruction is favoured by a hyper-contractile state, it would be logical to suppose that mavacamten may also be effective in patients with mid-ventricular obstruction (MVO). We present our experience with two HCM patients having MVO effectively treated with mavacamten.

**Case summary:**

The first case is a 55-year-old woman presenting with dyspnoea and exertional fatigue, with obstructive HCM (HOCM) and mid-ventricular peak gradient of 77 mmHg associated with LVOT obstruction. The treatment with mavacamten 5 mg daily determined relief of symptoms. At 16-week follow-up, there was a significant reduction of peak gradient (11 mmHg in mid-ventricular tract) and a significant decrease in NT-proBNP levels from 1287 to 178 ng/L. The second case is a 55-year-old woman with predominant mid-ventricular HOCM (peak gradient 52 mmHg) and past history of septal myectomy, with a residual significant gradient measured at LVOT level. The patient was started on mavacamten 5 mg daily, subsequently up-titrated to 10 mg. At 16-week follow-up, there was a significant reduction of peak gradient to 10 mmHg and a significant decrease in NT-proBNP levels from 3910 to 718 ng/L.

**Discussion:**

These two cases highlight the efficacy of mavacamten in the reduction of MVO, suggesting that it may be a valid therapeutic option also in patients with isolated MVO, frequently more difficult to be adequately treated.

**Learning points**

- Patients with hypertrophic cardiomyopathy and left ventricular mid-ventricular obstruction usually have a suboptimal response to available pharmacological treatments and are at higher risk of apical aneurysm and ventricular dysfunction.
- We present two patients with hypertrophic cardiomyopathy with left ventricular mid-ventricular obstruction documented by Doppler and three-dimensional echocardiography that was effectively relieved with mavacamten.
- Therapy with mavacamten in hypertrophic cardiomyopathy patients with isolated mid-ventricular obstruction may represent a valid therapeutic option.

## Introduction

Mavacamten is a cardiac-specific myosin inhibitor approved for the treatment of adults with symptomatic hypertrophic obstructive cardiomyopathy (HOCM).^1^ It has been developed to target the hyper-contractile phenotype, which is a crucial aspect of hypertrophic cardiomyopathy (HCM) pathophysiology. Mavacamten has shown in clinical trials a sustained reduction in left ventricular outflow tract (LVOT) gradients and improvement in exercise capacity and symptoms^2–4^. Therefore, it has been approved by the Food and Drug Administration and the European Medicines Agency for the treatment of adult symptomatic HOCM patients in New York Heart Association (NYHA) classes II–III. Both European and American guidelines for the management of HCM patients have recommended myosin inhibitors in the treatment of HOCM (classes IIA and IB, respectively).^5,6^

Left ventricular (LV) mid-cavity obstruction occurs in ∼10% of patients with HCM.^7^ Patients with mid-ventricular obstruction (MVO) tend to be very symptomatic and have an increased risk of developing apical aneurysms, progressive heart failure, and sudden cardiac death (SCD).^7^ Patients with HCM and MVO commonly receive high-dose beta-blockers, verapamil, or diltiazem, but the response to treatment is often suboptimal. Limited experience, mostly from single centres, suggests that MVO can be relieved by transaortic or transapical myectomy approach, but with uncertain long-term survival and greater complications rate.^8^ Published mavacamten trials enrolled HCM patients in the presence of dynamic obstruction documented by peak LVOT gradient. The coexistence of MVO was not an exclusion criteria in these trials,^2,3^ but results on MVO were not reported. Therefore, the effects of myosin inhibitors in symptomatic HOCM patients with isolated MVO are not yet established.

We present herein two HCM patients followed in our cardiomyopathy outpatient clinic in whom we document the relief of MVO after mavacamten treatment.

## Summary figure

Reduction of mid-ventricular gradient after 16 weeks of treatment in obstructive hypertrophic cardiomyopathy (HCM). On the left side is represented the 2DE, Doppler, and 3DE findings regarding MVO before and after mavacamten treatment in our first patient. (*A* and *B*) Colour Doppler showing mid-ventricular acceleration before and after mavacamten treatment; (*C* and *D*) 3DE showing systolic obliteration of LV cavity at mid-ventricular level relieved after mavacamten; (*E* and *F*) Mid-ventricular peak gradient relieved after mavacamten. On the right side are represented with the same findings regarding our second patient. (*G* and *H*) Colour Doppler showing mid-ventricular acceleration before and after mavacamten treatment; (*I* and *L*) 3DE showing systolic obliteration of LV cavity at mid-ventricular level relieved after mavacamten; (*M* and *N*) Mid-ventricular peak gradient relieved after mavacamten.

**Figure:**
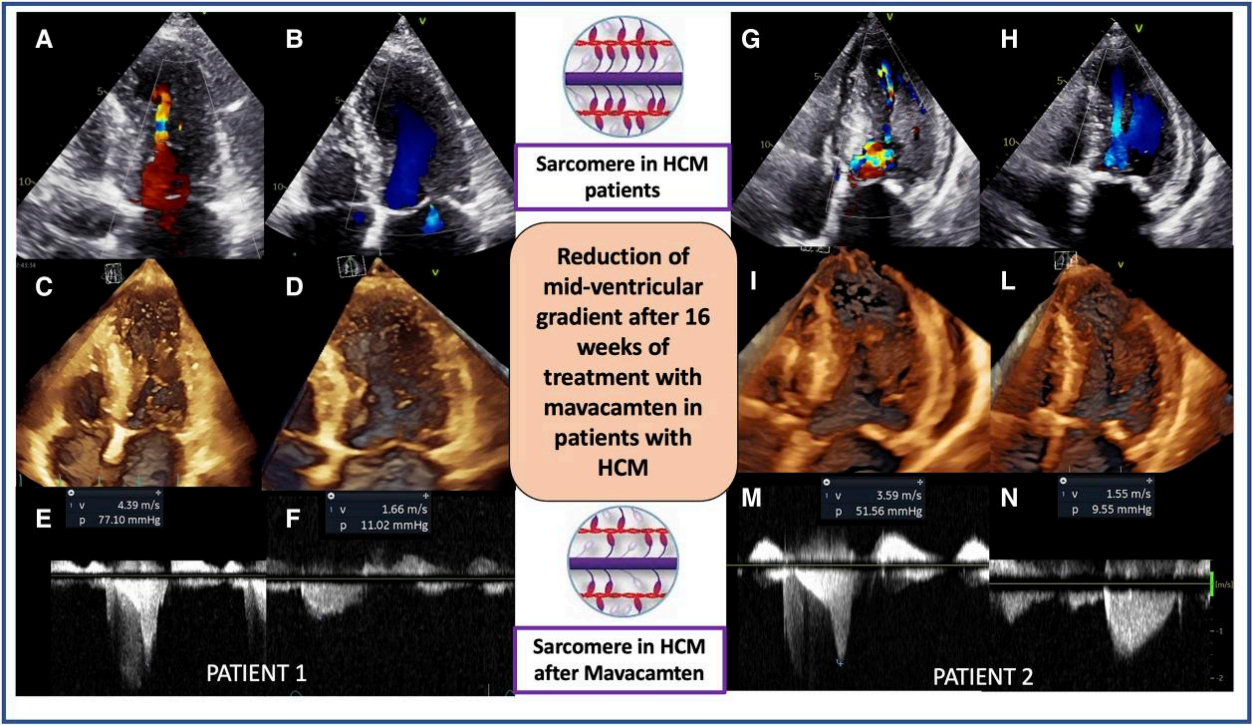


## Case summary

### Patient 1

The first case is a 55-year-old woman with HOCM, presenting with dyspnoea (NYHA classes II–III), exertional fatigue, and palpitations. Physical examination showed no signs of congestion, and a grade 3/6 systolic murmur was present. Her genetic test showed a variant of uncertain significance (VUS) on *MYLK2*. She was receiving treatment with bisoprolol (8.75 mg/day) and could not tolerate disopyramide. The electrocardiogram (ECG) showed sinus rhythm, high QRS voltage, Q waves, deep T-wave inversion, and ST depression in the inferior and lateral leads. Blood tests showed elevated NT-proBNP levels [1287 ng/L, normal values (n.v.) < 400 ng/L].

The cardiopulmonary exercise test showed a good functional capacity with VO2 at peak exercise of 21.4 mL/min/kg (90% of predicted) and VE/VCO2 slope of 35.42.

Echocardiography showed a mildly dilated LV (LV end-diastolic volume = 80 mL/m^2^, normal range 40–78 mL/m^2^), with asymmetrical septal LV hypertrophy (maximal wall thickness 22 mm, normal range 6–9 mm). The LV ejection fraction (LVEF) was 72% (normal range 54%–74%), without evidence of regional wall motion abnormalities and with preserved global longitudinal strain (GLS) (−18%, normal range between −17 and −25). Moreover, there was moderate mitral regurgitation.

**Figure 1:**
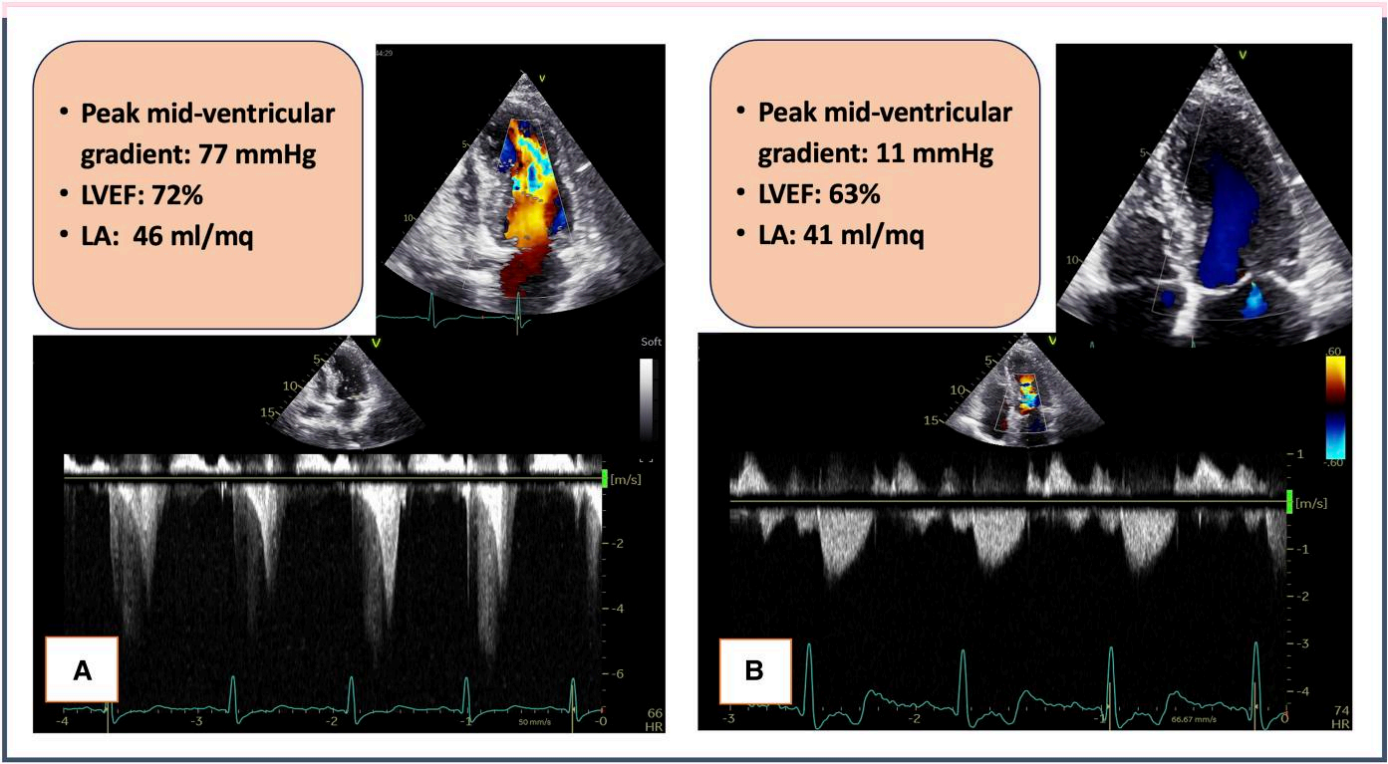
Echocardiographic characteristics at baseline and at 16 weeks follow-up in Patient 1. On the left side, baseline echocardiographic data are shown (*A*) while on the right side, data after 16 weeks of treatment with mavacamten are indicated (*B*). Note the differences in scale. LVEF, left ventricular ejection fraction; LA, left atrium.

Two-dimensional echocardiography (2DE) with colour Doppler was suggestive of MVO, which was confirmed by three-dimensional echocardiography (3DE) confirming systolic obliteration of LV cavity at mid-ventricular level (*Summary figure*, *C*). Septal and papillary muscle hypertrophy were responsible for MVO with peak LV dynamic gradient of 77 mmHg after Valsalva manoeuvre. A cardiac magnetic resonance study could not be performed due to specific contraindication (presence of a cochlear implant) (*Table 1* and *Figure 1A*).

**Table 1:** Clinical and echocardiographic characteristics of Patient 1 at baseline–4–8–12 and 16 weeks follow-up

Patient 1	Variables	Baseline	4 weeks	8 weeks	12 weeks	16 weeks
Mavacamten dose			5 mg/day	5 mg/day	2.5 mg/day	2.5 mg/day
Clinical and echo	NYHA class	II–III	II	II	II	I
	Maximum wall thickness (mm)	22	20	19	20	19
	Left atrial volume (mL/mq)	46	39	45	42	41
	Peak mid-cavity gradient (mmHg)	77	10	5	5	11
	Left ventricular ejection fraction (LVEF %)	72	60	53	63	63
	LV global longitudinal strain (GLS) %	−18	−16.9	−15.7	−18	−17
	E/e′	10	9.8	11.7	8	8
	Systolic anterior motion (SAM)	Yes	No	No	No	No
	Mitral regurgitation (grade)	++	+	+	+	+
Biomarkers	NT-proBNP (ng/L)	1287	–	–	–	178
Medical therapy	Bisoprolol (mg)	8.75	6.25	6.25	6.25	5

The treatment with mavacamten 5 mg/day was initiated, due to the coexistence of a peak gradient measured by CW Doppler at LVOT level ≥ 30 mmHg on top of her current medications. During the ambulatory check-ups, she reported an immediate relief of the dyspnoea, even though she still had fatigue and tachycardia. The echocardiographic control at 8-week visit showed a reduction of the peak gradient (11 mmHg) and of the LVEF measured by 3DE (LVEF = 53%). Due to persistent symptoms, reduction of LVEF (but not below 50%) and LV peak gradient below 20 mmHg, mavacamten dose was reduced to 2.5 mg daily. At 16-week follow-up, the patient reported symptomatic improvement (NYHA I) with resolution of weakness, associated with improvement in LV systolic function (3D LVEF 63%), complete abolition of LV obstruction, as well as mild improvement in LV diastolic function parameters [E/e′ = 8, left atrium (LA) maximal volume = 41 mL/m^2^] (*Table 1* and *Figure 1B*).

### Patient 2

The second case regards a 55-year-old woman diagnosed with severe HOCM who underwent previous surgical septal myectomy and tricuspid valve annuloplasty with a residual peak gradient measured by CW Doppler at LVOT level ≥ 30 mmHg allowing mavacamten treatment. Physical examination showed no signs of pulmonary congestion or peripheral oedema, and a grade 2/6 systolic murmur was audible. A transvenous defibrillator was implanted for primary prevention, and she was treated with nadolol (120 mg daily). After surgery, she complained of dyspnoea (NYHA class III) associated with exertional fatigue. Her genetic test showed a VUS on *MYH7*. The ECG showed sinus rhythm with a left bundle branch block. The NT-proBNP levels were elevated (3910 ng/L).

**Figure 2:**
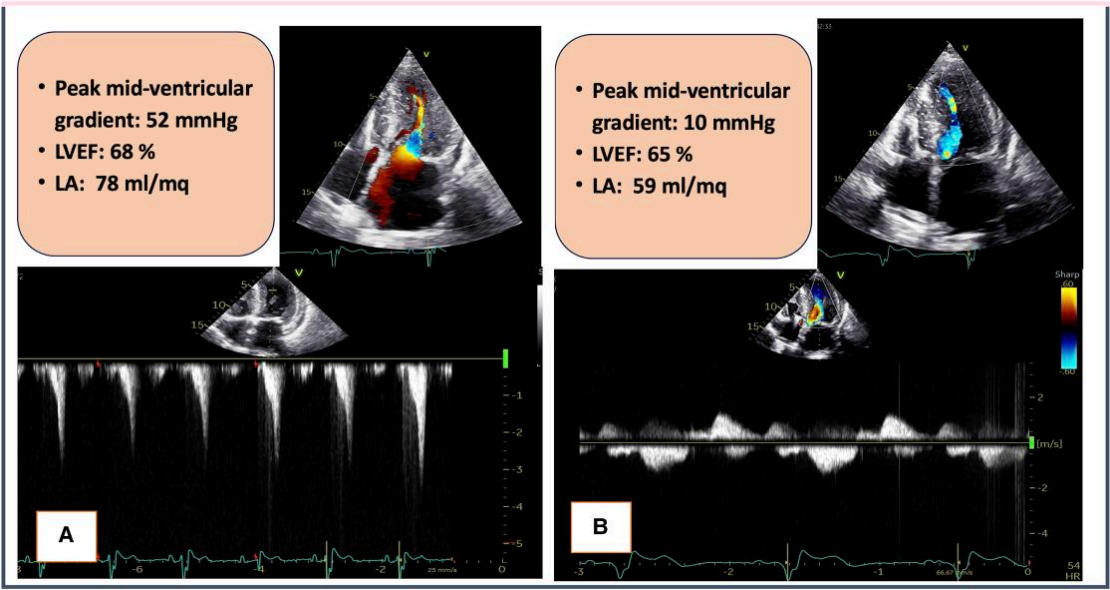
Echocardiographic characteristics at baseline and at 16 weeks follow-up in Patient 2. On the left side baseline echocardiographic data are shown (*A*) while on the right side, data after 16 weeks of treatment with mavacamten are indicated (*B*). Note the differences in scale. LVEF, left ventricular ejection fraction; LA, left atrium.

Echocardiography showed a dilated LV (LV end-diastolic volume = 107 mL/m^2^), with a residual mid septal wall thickness of 28 mm and papillary muscle hypertrophy leading to MVO (peak gradient = 52 mmHg). LV ejection fraction measured by 3DE was normal (LVEF = 68%), associated with impaired LV longitudinal myocardial deformation assessed by 2DE speckle-tracking (GLS = −16%). There was a moderate mitral regurgitation, signs of elevated LV filling pressures (E/e′ = 27), severe left atrial dilation (LA maximal volume = 78 mL/m^2^), and moderate pericardial effusion (10 mm) (*Table 2* and *Figure 2A*).

**Table 2:** Clinical and echocardiographic characteristics of Patient 2 at baseline-4-8-12 and 16 weeks follow-up

Patient 2	Variables	Baseline	4 weeks	8 weeks	12 weeks	16 weeks
Mavacamten dose			5 mg/day	5 mg/day	5 mg/day	10 mg/day
Clinical and echo	NYHA class	III	II	II	II	II
	Maximum wall thickness (mm)	28	28	27	27	26
	Left atrial volume (mL/mq)	78	82	77	69	59
	Peak mid-cavity gradient (mmHg)	52	51	37	55	10
	Left ventricular ejection fraction (LVEF %)	68	66	60	69	65
	LV global longitudinal strain (GLS) %	−16	−16.8	−14.5	−16.8	−16
	E/e′	27	22	24	26	19
	Systolic anterior motion (SAM)	Yes	Yes	Yes	Yes	No
	Mitral regurgitation (grade)	+++	+/++	+	+/++	+/++
Biomarkers	NT-proBNP (ng/L)	3910	–	–	–	718
Medical therapy	Nadolol (mg)	120	120	120	120	80

Stress echocardiography and cardiopulmonary exercise testing showed a severe reduction of functional capacity and chronotropic incompetence (maximal heart rate 80 b.p.m., 48% of theoretical heart rate) with a VO2 peak of 13 mL/kg/min (59% of predicted), and severe mitral regurgitation with mid-cavity peak gradient of 90 mmHg.

The patient was started on mavacamten 5 mg daily that was up-titrated to 10 mg after 12 weeks due to persistent dynamic gradient > 20 mmHg. At the 16-week follow-up, she reported symptomatic improvement (NYHA I–II), associated with a reduction of NT-proBNP levels to 718 ng/L.

Echocardiography showed a resolution of gradient, as well as mild improvement in LV diastolic function parameters (E/e′ = 19, LA maximal volume = 59 mL/m^2^) (*Figure 2B*).

## Discussion

These two cases illustrate the efficacy of mavacamten in treating MVO in HCM patients.

Mid-ventricular obstruction is usually related to LV hyperkinesia in the presence of a hypertrophied mid-septum and hypertrophied papillary muscles. The MVO is distinct from LVOT obstruction and from SAM of the mitral valve. Colour Doppler shows turbulence at the mid-ventricular level, and the continuous wave Doppler profile is typically depicting a late-peaking systolic velocity (*Summary figure*). Mid-ventricular obstruction may be present with or without apical aneurysm formation.

A recently published case report^9^ showed an unusual case of HOCM with predominant right ventricular outflow tract obstruction and concomitant LVOT and mid-ventricular obstruction documented by cardiac catheterization in which mavacamten effectively reduced all gradients. The mechanism of action of mavacamten in MVO is likely the same as for the reduction of left ventricular outflow obstruction, i.e. the reduction of the hyper-contractile state of the ventricle. This observation is clinically relevant, as HCM patients with MVO usually have a suboptimal response to available pharmacological treatments. Moreover, they are at higher risk of complications during myectomy, which is also usually less effective. If not adequately treated, HCM patients with MVO tend to develop apical aneurysm and LV dysfunction that together increase the risk of both heart failure and SCD. As mavacamten can reduce LVEF and large apical aneurysms by themselves have a negative impact on LV systolic performance, therapy with mavacamten in patients with MVO should probably be started as early as possible, to prevent the aneurysm formation.

We have also documented that transthoracic 3DE enables the visualization of the anatomic obstruction; in addition, it allows an accurate quantification of LV volumes and ejection fraction. *Summary figure* illustrates the 2DE, Doppler, and 3DE findings regarding MVO before and after mavacamten treatment in our patients.

The clinical observations reported, combined with the pathophysiological reasoning, suggest that mavacamten may also be a suitable therapeutic option in patients with isolated MVO due to hypertrophy of the septum and papillary muscle, not related to the SAM phenomenon.

Recognizing MVO and multilevel obstruction (MVO coexisting with LVOT obstruction) by a thorough and careful echocardiographic exam has important implications. For instance, alcohol septal ablation should not be utilized in these cases and surgery should be preferred. However, failure to recognize a significant associated MVO may lead to suboptimal gradient reduction after surgical myectomy of the basal septum, as in our first patient.^10^ A trial of mavacamten in HCM patients without LVOT obstruction (ODYSSEY-HCM, NCT05582395; clinicaltrials.gov) and a trial on aficamten (ACACIA-HCM, NCT06081894; clinicaltrials.gov), the other myosin inhibitor under investigation, on the same group of patients (also including isolated MVO) are in progress. These trials will probably clarify in the near future the role of myosin inhibitors in patients with isolated MVO.

## Lead author biography


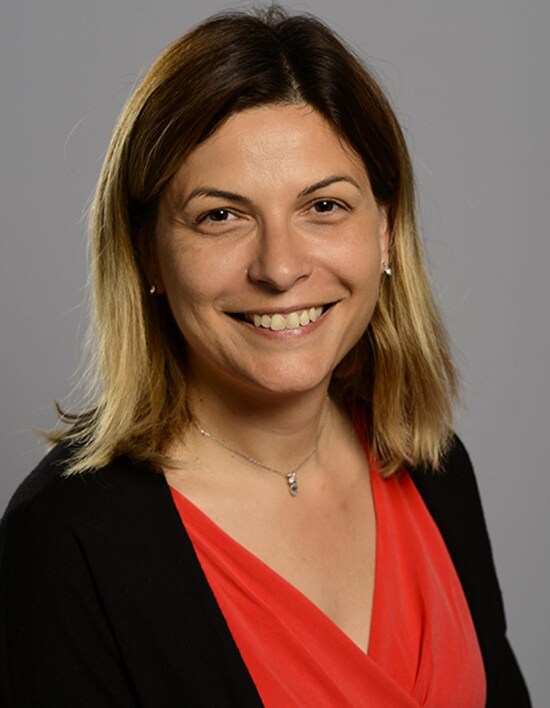


Lia Crotti is associate professor of Cardiology at the University Milano Bicocca in Milan and she is the director of the Cardiomyopathy Unit and Rehabilitation Unit of the IRCCS Istituto Auxologico Italiano in Milan, Italy. Furthermore, she is vice-director of the Center for Cardiac Arrhythmias of Genetic Origin and Laboratory of Cardiovascular Genetics of the same Institute. Lia Crotti is an internationally renowned expert in channelopathies and cardiomyopathies and her research interests are mainly focused on the genetic basis of sudden cardiac death. Furthermore, her research is focused on the improvement of the clinical management and treatment of patients affected by arrhythmogenic diseases.

## Data Availability

The data underlying this article are already available in the text of this article. Additional data are available upon request.

## Notes

**Consent:** The patients gave their informed consent in line with COPE guidelines for the present manuscript.

**Funding:** This work was funded by Italian Ministry of Health.

## References

[1] Braunwald E, Saberi S, Abraham TP, Elliott PM, Olivotto I. Mavacamten: a first-in-class myosin inhibitor for obstructive hypertrophic cardiomyopathy. Eur Heart J 2023;44:4622–4633. 37804245 10.1093/eurheartj/ehad637 PMC10659958

[2] Olivotto I, Oreziak A, Barriales-Villa R, Abraham TP, Masri A, Garcia-Pavia P, et al Mavacamten for treatment of symptomatic obstructive hypertrophic cardiomyopathy (EXPLORER-HCM): a randomised, double-blind, placebo-controlled, phase 3 trial. Lancet 2020;396:759–769. 32871100 10.1016/S0140-6736(20)31792-X

[3] Desai MY, Owens A, Geske JB, Wolski K, Naidu SS, Smedira NG, et al Myosin inhibition in patients with obstructive hypertrophic cardiomyopathy referred for septal reduction therapy (VALOR HCM). J Am Coll Cardiol 2022;80:95–108. 35798455 10.1016/j.jacc.2022.04.048

[4] Hegde SM, Lester SJ, Solomon SD, Michels M, Elliott PM, Nagueh SF, et al Effect of mavacamten on echocardiographic features in symptomatic patients with obstructive hypertrophic cardiomyopathy. J Am Coll Cardiol 2021;78:2518–2532. 34915982 10.1016/j.jacc.2021.09.1381

[5] Arbelo E, Protonotarios A, Gimeno JR, Arbustini E, Barriales-Villa R, Basso C, et al 2023 ESC Guidelines for the management of cardiomyopathies. Eur Heart J 2023;44:3503–3626. 37622657 10.1093/eurheartj/ehad194

[6] Ommen SR, Ho CY, Asif IM, Balaji S, Burke MA, Day SM, et al 2024 AHA/ACC/AMSSM/HRS/PACES/SCMR. Guideline for the management of hypertrophic cardiomyopathy: a report of the American Heart Association/American College of Cardiology Joint Committee on Clinical Practice Guidelines. Circulation 2024;149:e1239–e1311. 38718139 10.1161/CIR.0000000000001250

[7] Minami Y, Kajimoto K, Terajima Y, Yashiro B, Okayama D, Haruki S, et al Clinical implications of midventricular obstruction in patients with hypertrophic cardiomyopathy. J Am Coll Cardiol 2011;57:2346–2355. 21636036 10.1016/j.jacc.2011.02.033

[8] Said SM, Schaff HV, Abel MD, Dearani JA. Transapical approach for apical myectomy and relief of midventricular obstruction in hypertrophic cardiomyopathy. J Card Surg 2012;27:443–448. 22640263 10.1111/j.1540-8191.2012.01475.x

[9] Malhi JK, Carrick RT, Duvall C, Rahman F, Martinez MW, Madrazo J, et al Mavacamten in right ventricular outflow tract obstruction. JACC Case Rep 2024;29:102397. 38952423 10.1016/j.jaccas.2024.102397 PMC11215104

[10] Abbasi M, Ong KC, Newman DB, Dearani JA, Schaff HV, Geske JB. Obstruction in hypertrophic cardiomyopathy: many faces. J Am Soc Echocardiography 2024;37:613–625. 10.1016/j.echo.2024.02.010 38428652

